# Applying Design Thinking for Co-Designed Health Solutions: A Case Study on Chronic Kidney Disease in Regional Australia

**DOI:** 10.3390/ijerph22101475

**Published:** 2025-09-24

**Authors:** Anita Stefoska-Needham, Jessica Nealon, Karen Charlton, Karen Fildes, Kelly Lambert

**Affiliations:** 1 School of Medical, Indigenous and Health Sciences, University of Wollongong, Wollongong 2522, Australia; jnealon@uow.edu.au (J.N.); karenc@uow.edu.au (K.C.); kfildes@uow.edu.au (K.F.); klambert@uow.edu.au (K.L.); 2 School of Health Sciences, University of New South Wales, Sydney 2052, Australia

**Keywords:** design-led thinking, codesign, consumer, chronic kidney disease, qualitative research

## Abstract

(1) Background: This paper outlines key issues to consider when implementing Design Thinking methodology in health-based qualitative research to achieve a meaningful outcome. The purpose is to share our learnings with others. (2) Methods: Using the case study of an Australian region with high rates of chronic kidney disease, we describe a design-led methodological approach (co-design) that ensures end users remain central to research for the lifespan of the project; from conception of the research question and protocol design, through to solution generation and change implementation. (3) Results: Representation of the four Design Voices—people with lived experience, expertise, intent, and design knowledge—was imperative to minimise bias towards researchers as the main drivers of the project. A commitment to the five core elements of design thinking (empathising, defining, ideating, prototyping, and testing) was maintained throughout the research. Empathising through direct interaction with users was crucial to creating a meaningful understanding of their problems and challenges. Ideation ensured user-centred solution generation, with solutions aligned with addressing the ‘real’ problem and creating an improved future state. (4) Conclusions: Incorporation of Design Thinking principles in health research is a valuable adjunct to traditional qualitative methodologies, with the potential to facilitate meaningful outcomes for people in our community experiencing a wicked health problem.

## 1. Introduction

Chronic kidney disease (CKD) poses a significant public health burden in Australia [1]. In the Illawarra and Shoalhaven region of New South Wales, the prevalence of CKD is around 9% [2]. In 2018, researchers in the Health Impacts Research Centre (HIRC) at the University of Wollongong, New South Wales, embarked on a multidisciplinary research programme to investigate reasons for the high rates of CKD, and to design potential interventions to prevent or slow the progression of the disease in the region. Community engagement was recognised as an important starting point in this endeavour, the specific aim of which was to better understand the diverse experiences of people living with CKD, their family members, carers, and health care providers. To achieve this aim, researchers within HIRC were committed to implementing an *authentic* co-design process that was underpinned by the principle of ‘design thinking’, a lesser-known approach among academic health researchers generally, but more broadly recognised in business, technology, and service design sectors. Design Thinking, also known as design-led thinking, emerged prominently in these fields as a structured yet flexible approach for addressing complex challenges [3]. Its emphasis on empathy, ideation, and iterative problem-solving has been widely adopted to improve user experiences and drive organisational change. More recently, health and social care research has begun to adopt design-led principles, recognising their potential to address multifaceted or “wicked” problems that resist simple solutions. Unlike traditional top-down research approaches, design-led thinking is explicitly participatory and user-centred, positioning lived experience as a critical form of evidence alongside clinical or academic expertise [4,5]. This framing helps to bridge the gap between health service users and providers by generating solutions that are not only theoretically desirable but also practically feasible and economically viable [6]. In the context of chronic conditions such as CKD, where care pathways are fragmented and experiences vary widely, design-led thinking can uncover hidden barriers and co-create person-centred innovations that align with the realities of diverse stakeholders.

Therefore, using the complex problem of high CKD rates in the Illawarra and Shoalhaven regions of Australia as an example, this viewpoint paper outlines some key issues to consider when implementing design thinking methodology in health-based qualitative research. We intend to share our learnings with the broader academic health services research community so that others may also successfully use codesign. The concept of design thinking is defined, and its potential value in research that explores a complex health problem in the community is showcased. Suggestions for successfully incorporating design thinking into traditional qualitative research methodologies are also made. Finally, we suggest that Design Thinking is elevated from being considered as a technology/innovation-specific tool for solution generation to a valuable adjunct to traditional qualitative research methodologies, with the potential to facilitate meaningful outcomes for people in our community experiencing a multifarious health problem.

## 2. Materials and Methods

### 2.1. Overview of Design Thinking 

**Figure 1:**
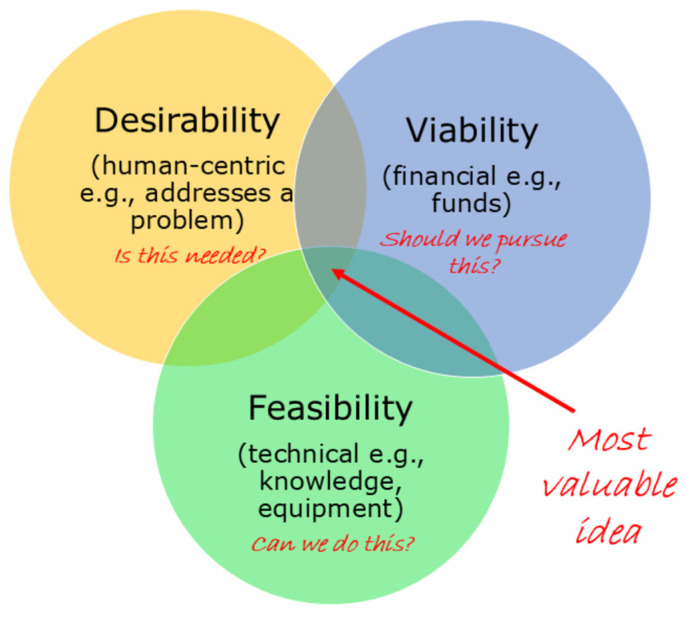
The three design lenses: desirability, viability, and feasibility to guide the selection of the most valuable idea or solution (adapted from [7]). The most valuable idea or solution to a problem is the one that meets all three criteria.

Design Thinking encompasses an iterative and human-centred approach to solving complex problems [3] by promoting empathy for those experiencing the problem, and thereby encouraging the creation of human-centred, also known as user-centred, solutions [4,5]. Traditionally, the Design Thinking approach has been successfully implemented within technology and business sectors to enhance products and improve services, and relies strongly on collaborative multidisciplinary teams working together with the end users to achieve desired outcomes, or to design a valuable solution [6]. These “users” are generally people in the community who may engage in a product or service, such as customers. In employing Design Thinking, establishing the nexus between what is desirable from a human perspective with what is technologically feasible and economically viable, is pivotal because the most valuable idea or solution to a problem is the one that meets all three criteria [7] (Figure 1). This methodological approach, often described as ‘design-led’, reflects the underpinning Design Thinking principles. 

**Figure 2:**
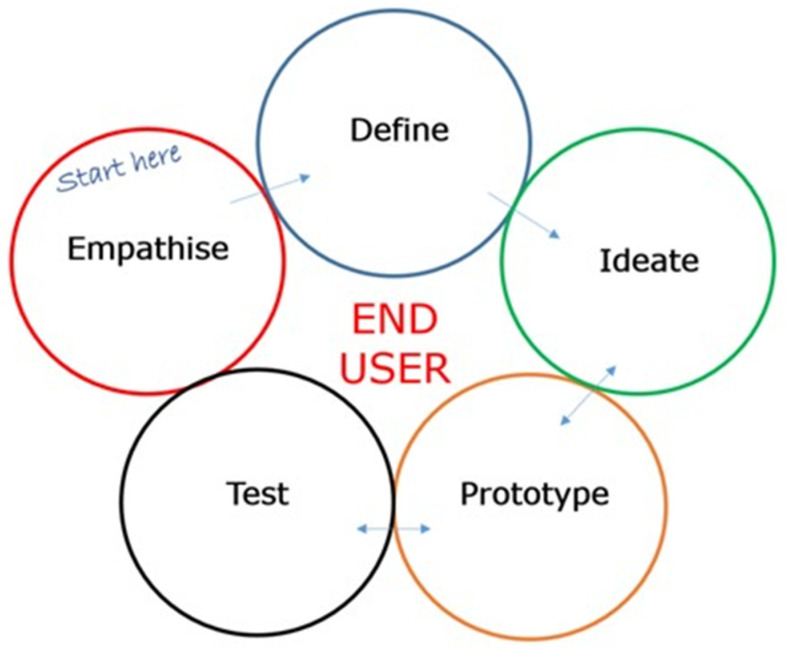
The five phases of Design Thinking, adapted from Stanford University’s Design Thinking Model: a strategy for authentic and innovative user-centric solution generation [4].

Typically, this process requires repeated cycles of brainstorming with all key stakeholders, including end-users, to generate ideas for a potential solution, followed by testing to identify potential barriers, which then may feed back into further ideation. Eventually, through this iterative process, the most valuable solution or idea is generated [8,9]. Tom Brown of Stanford University, one of the seminal proponents of Design Thinking, proposes that solution generation through Design Thinking is achieved across five stages: (i) empathising, (ii) defining, (iii) ideating, (iv) prototyping, and (v) testing [4] (Figure 2). 

**Figure 3:**
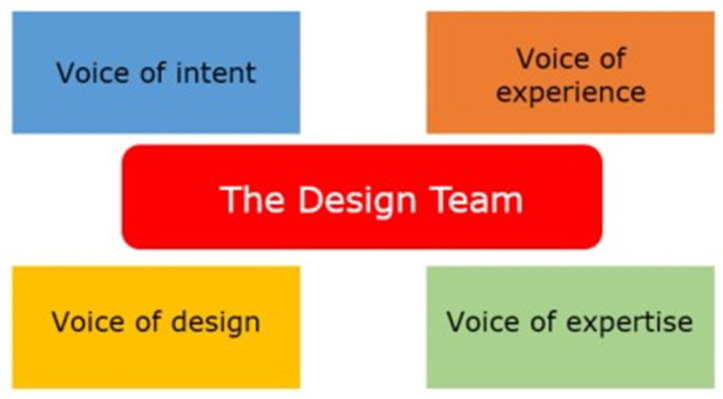
The Design Voices (adapted from and used with permission from ThinkPlace Global, [11]) represent four key groupings of stakeholders which are important for the design and execution of an effective co-design project.

Empathising relates to direct interaction with users, as an instinctive, emotional, affective, shared, and mirrored experience [5] crucial to making sense of things and creating meaning [10]. It helps to understand a need or a problem from the perspective of the end-user. The “define” phase allows reflection on the articulated need or problem in the “empathise” phase, as a means to devise a plan for a solution. The “ideation” phase includes creative brainstorming and innovative solution generation, and may include research on how to translate the solution into reality. During the “prototype” phase, an authentic and tangible solution is brought to life, often through numerous, rapidly generated user-centred prototypes. In the “test” phase, review and feedback of the prototype are received to ensure it meets the end-users’ needs, and includes iterations and refinements to the prototype, resulting in its final implementation or launch. According to Brown [4], to ensure that design-led activities are authentic requires the representation of four Design Voices throughout the process: the voice of intent (senior stakeholders), the voice of experience (the users), the voice of expertise (domain experts), and the voice of design (external facilitator guiding the process authentically) (Figure 3). Representation of all four Design Voices throughout the project minimises bias towards one voice over another.

From a pragmatic perspective, a number of tools are commonly used to collect data in design-led projects, including workshops, interviews, surveys, storyboarding, personas, and journey maps, depending on the needs and preferences of the users involved. For example, personas are fictional characters that are created based on research data, and are useful to embed empathy and to create an improved experience for the target group [12,13]. They are widely used in product design to create reliable and realistic representations of a key audience or target group for reference [14], but are also emerging in traditional qualitative research [15]. A journey map is a visual representation of a persona’s experiences and interactions over time, and helps to develop an understanding of how these experiences and interactions may be improved [16].

For many years, Design Thinking has also been used for social innovation, across different community segments to solve complicated and complex problems (also referred to as wicked problems) [17]. Globally, governments and private businesses alike have recognised the value of engaging with the community to design services and products that involve people who use or are affected by that service or product [18]. In health care systems, Design Thinking has also been used to restructure patient-oriented clinical services, aiming to improve patient journeys and clinical outcomes [19,20,21].Typically, the process of implementing Design Thinking methodologies is referred to as “co-design” in the health research literature [8]. Stakeholders involved in redesigning health system-related processes may include patients and their families and carers, clinicians, nursing staff, executives, and operational managers [22]. By coming together to co-design a solution, multi-stakeholder perspectives are represented, with no one voice being more dominant or important than the other. Chronic kidney disease (CKD) is a complex, even wicked, public health problem in Australia [1] and is well-suited to a design-led approach in finding a solution to the broad health and economic challenges it presents to communities. CKD refers to all conditions of the kidney, lasting at least three months, where there is evidence of kidney damage and/or reduced kidney function, regardless of the specific diagnosis of disease or condition causing the disease [23]. CKD is associated with an increased risk of morbidity and mortality [24], as well as significant economic costs, as high as $9.9 billion Australian dollars per year. Of concern is the overrepresentation of CKD amongst the Aboriginal and Torres Strait Islander community [1]. CKD is a multifaceted condition that not only affects multiple metabolic pathways [23], but it has impacts on the mental health of patients [25] and is usually associated with considerable co-morbidity and ageing [26]. This complexity makes CKD difficult to manage, both from the perspective of the patient, the patient’s family/carers, as well as health care providers.

Person-centred health care is recommended for the management of CKD due to the multifactorial nature of the condition; however, there is evidence to suggest that this standard is not being achieved consistently across Australia [21]. Person-centred health care is defined as “health care that involves the patient, their carer and family; and is respectful of and responsive to the preferences, needs and values of patients and consumers” [27]. Shortfalls in the delivery of CKD person-centred health care in Australia have emerged through reported gaps in treatment targets and failures to meet other recommended health indicators of optimal clinical care, such as treatment of anaemia [28]. Missed or late diagnosis of CKD and late referral of at-risk patients to nephrologists have also been documented [29]. Patient activation, described as an individual’s knowledge, skills, and confidence for managing their health and health care [30], is an essential component of the successful delivery of person-centred health care. In people with CKD, low levels of patient activation have been reported, especially in patients with co-morbidities, older patients, and those with *worse* self-reported health [31,32]. Low patient activation is associated with poor participation and engagement in an individual’s own health care, which challenges the core pillars of the person-centred health care philosophy. 

Integrated care is also important for the management of CKD. This approach has been shown to have favourable impacts on people’s self-management and their short-term clinical outcomes, as well as on CKD progression over time [33,34,35]. Key benefits of integrated care include improved communication between health professionals, clarity among primary care practitioners around guidelines, referral to specialist care, and more time available for individualised education [36]. Neale et al. [37] identified provider-related barriers to integrated care, such as confusion with clinical guidelines, inadequate financial reimbursement for screening, and lack of supportive primary care technology. Hence, the redesign of CKD services must consider integrative care as a core activity or philosophy. 

### 2.2. Community Engagement Is Essential in the Design of Person-Centred Health Care for People Living with CKD

To better achieve person-centred health care and thereby increase patient activation in CKD, it is important to meaningfully engage the community affected by CKD, including patients, carers, family, and health care practitioners (and numerous other stakeholders). This holistic approach has greater potential to offer a better understanding of existing challenges and help to identify opportunities for improvement. From a health perspective, co-design (underpinned by Design Thinking) is a way of “improving health care services for patients by bringing together all stakeholders and consumers in partnership, to develop health services that best meet the needs of consumers and carers in the most effective way possible” [38]. The commitment to and support of co-design in health is also reflected at the state health department level with the formation of the Agency for Clinical Innovation (ACI) (NSW, Australia), commissioned to engage patients and consumers to design, promote and implement a person-centred approach to health care in NSW using co-design methodologies [38]. Recently, kidney care services in Victoria and New South Wales have been co-designed with people with CKD, their families/carers, and health practitioners [21], with potential for nationwide roll-out, though this is yet to be confirmed. 

## 3. Results

In the Health Impacts Research Centre (University of Wollongong, Wollongong, NSW, Australia) CKD project [39], community engagement was identified as a key strategic research priority. Thus, a specific community engagement hub was created that included researchers skilled in qualitative research, key stakeholders working in CKD from the Local Health District, members of the community, and other stakeholders, according to Design Thinking principles. The results are published elsewhere [39]. The community engagement hub worked alongside a hub of researchers conducting epidemiological research, as well as a hub of researchers performing systematic reviews of the literature, as part of a multi-disciplinary approach to understanding why CKD rates are high relative to other regions of Australia. 

**Figure 4:**
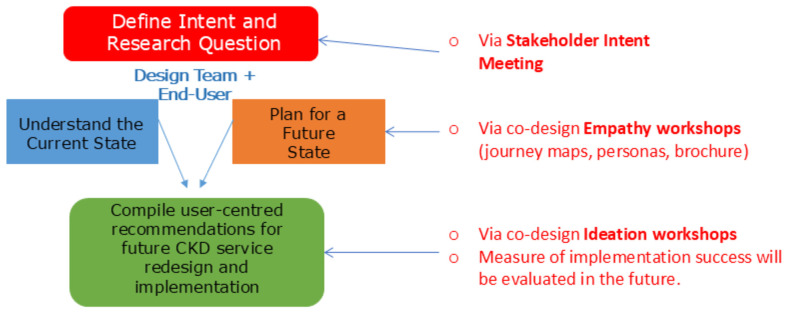
Overview of the CKD co-design project underpinned by Design Thinking methodologies, from defining the project intent to developing recommendations for the redesign of new CKD services in the Illawarra–Shoalhaven region.

The execution of the design-led HIRC CKD project followed a number of key steps reflecting the important principles of Design Thinking (see Figure 4). Stakeholders were assembled that reflected the four Design Voices, and an intent meeting was held to set expectations to guide the project for the duration of its lifecycle. The intent meeting included the facilitation of activities designed to (i) elicit insights from end users in an empathetic way that draws upon lived experiences to better define the current state and existing challenges, and (ii) offer users opportunities to ideate and propose (prototype) solutions to their perceived problem and challenges. 

The key steps are broadly described here:

*Step 1: Recruitment of Stakeholders to Represent the Four Design Voices*

**Figure 5:**
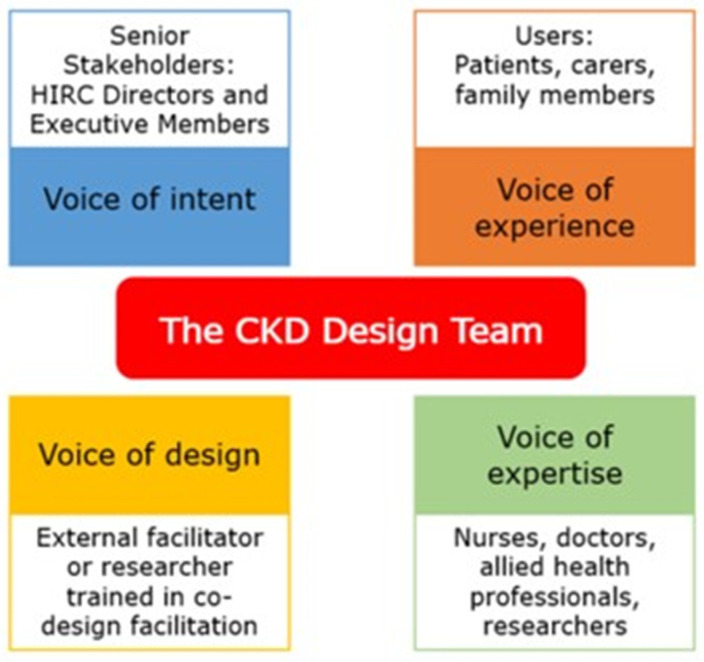
The Design Team, comprising key stakeholders representing the four Design Voices underpinning the HIRC CKD Project. The *voice of intent* was represented by HIRC directors, executive members, and project managers. The *voice of experience* was represented by the patients and their family/carers. The *voice of expertise* was represented by researchers and medical and allied health professionals. The *voice of design* was represented by an expert co-design facilitator.

For the recruitment of the four representative Design Voices in the HIRC CKD project, purposive sampling was employed. Purposive sampling is the intentional selection of participants based on their ability to provide detailed and extensive information about the phenomenon under investigation [40]. The voice of intent was represented by senior stakeholders, including HIRC directors (*n* = 2) and executive members (*n* = 5), as well as HIRC CKD project managers (*n* = 4). The voice of experience was represented by the patients (with a formal CKD diagnosis across the spectrum of disease stages), and their family/carers living in the Illawarra-Shoalhaven region. The participants were recruited from the Local Health District Renal Units and general medical practices, where study posters and flyers were showcased in consulting and waiting rooms to promote the project. The voice of expertise was represented by nurses, doctors, allied health professionals, alongside researchers (members of HIRC at the University of Wollongong with an interest in health research). Eligible secondary and tertiary health care practitioners were required to be actively practising as secondary or tertiary health care practitioners based in the Illawarra-Shoalhaven region. They were directly contacted by one of the HIRC CKD project researchers to seek their voluntary participation. Finally, the voice of design was represented by an external facilitator guiding the co-design process authentically. The ongoing representation of the four Design Voices throughout the project was crucial to remaining authentic to the co-design process and minimising bias. Figure 5 represents the four Design Voices overseeing the HIRC CKD project.

*Step 2: Defining the Project’s Intent, Problem Statement, and Setting Expectations*

Following the collation of the four Design Voices, the voice of intent representatives led a meeting to define the project’s intent, involving stakeholders representing all Design Voices. The intent of the project was defined as follows: “*to understand the current state for people living with CKD, their family and carers, in the Illawarra-Shoalhaven region, and use these insights to recommend changes to achieve a future state with improved patient/user journeys and more empathetic renal services that meet the complex needs of its varied users.*” To add rigour to the methodological approach, the appropriateness of the project’s intent was tested through the three lenses of Design Thinking: desirability (is the project needed by end users?/is it valuable?), is it viable (do we have the financial means to conduct the project activities?), is it feasible (do we have the experience and expertise to conduct the work?). For the purposes of this project, end users included people with CKD and their families/carers, as well as health care professionals working within Illawarra-Shoalhaven renal services. Stakeholders at the intent meeting deemed that all three Design Thinking criteria were satisfied, and hence, the project received consensus agreement for launch. 

The main value and output of the intent meeting was the design of a research question together with all relevant stakeholders, rather than researchers proposing the question exclusively. Traditional academic approaches to research design typically start with a predetermined research question that is seldom tested or validated with stakeholders, and is most certainly proposed in isolation from the collective Design Voices. The research question often emerges from identified gaps in the previously published literature or prior research activities. This approach is therefore unlikely to be an authentic reflection of co-design or design-led principles, as it is biased towards one stakeholder, that being the researcher [41]. This is therefore a major, though less acknowledged or recognised, limitation of current qualitative research purporting to be “co-designed”. 

The intent meeting also offered an early-stage opportunity to identify other stakeholders to include within the four Design Voices, to ensure the project maintained its authenticity to co-design and methodological rigour throughout the duration of the research period. Following the initial intent meeting, the research question and protocol underpinning the HIRC CKD project were formally drafted and validated with representatives of all the Design Voices, and approval from the University of Wollongong Human Research Ethics Committee was received (HE2018/348).

*Step 3: Empathising with the End Users to Collect Valuable Insights (Data) and Define the Problem, and Ideate Potential Solutions*

Through a series of workshops facilitated by the *voice of design* (external facilitator and trained HIRC researchers), insights from people with CKD, family members, carers, health researchers, and health care providers (representing both the *voice of experience* and *voice of expertise*) were conducted over a two-year period in 2018 and 2019. In addition, telephone interviews were conducted with some stakeholders who were unable to attend the workshops. The first workshops were called *Empathy Workshops* to reflect the purpose of building empathy with the users to better understand their experiences, and their perspectives on the current state and problem. The second round of workshops was called *Ideation Workshops*, which represented opportunities for users to ideate or brainstorm solutions. 

### 3.1. Empathy Workshops 

**Figure 6:**
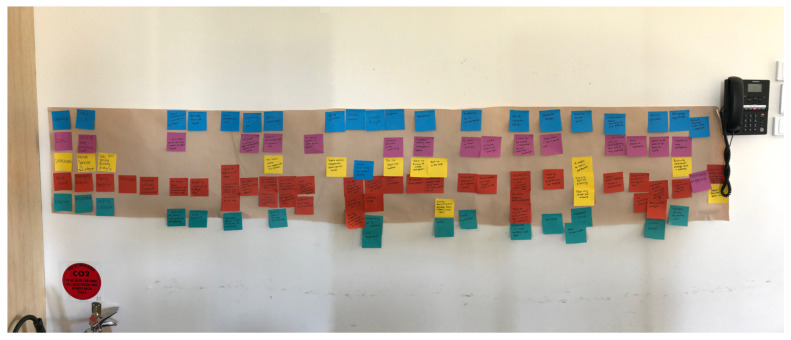
Image of Post-it notes generated by scribes with consumers during the co-design process. These notes represent the user perspectives and experiences, arranged on a wall chart to form user journeys. The notes are grouped according to four themes: narrative (or background story), user satisfaction points, challenges, and ideas for change.

For each small group discussion at the workshops, a ‘scribe’ was allocated to capture key points on Post-it notes (Figure 6). Each small group consisted of people with similar experiences, such as people on dialysis, people with a kidney transplant, or health professionals. Segmentation of groups enabled potential power imbalances to be minimised. The scribe was usually a HIRC researcher acting in their role as the voice of design, under the broader guidance of the external facilitator. These Post-it notes were then attached to a wall chart representing elements of a user journey through kidney care services in the Illawarra-Shoalhaven region. The user journey wall chart comprised four themes or subheadings: (i) narrative or background story; (ii) user satisfaction points; (iii) pain points or challenges; and (iv) ideas for change (see Figure 6). The users within each small group sorted the Post-it notes into the four categories. Photographs of each of the small groups’ wall charts were taken for further data analysis by researchers who conducted thematic analysis to identify, analyse, and report themes within the data [42]. Thematic analysis summarises and systemises the content of the qualitative data and is one of the most widely used approaches in health research [43]. As thematic analysis involves active researcher interpretation, reflexivity was used to enhance rigour and transparency [44]. This included regular self-reflection and team discussions to consider how researchers’ backgrounds and perspectives might shape data interpretation. Findings of this analysis, published in full elsewhere [39], indicate there are challenges with timely access to kidney care services and a need to dismantle health professional silos that lack good interdisciplinary communication. 

### 3.2. Personas

**Figure 7:**
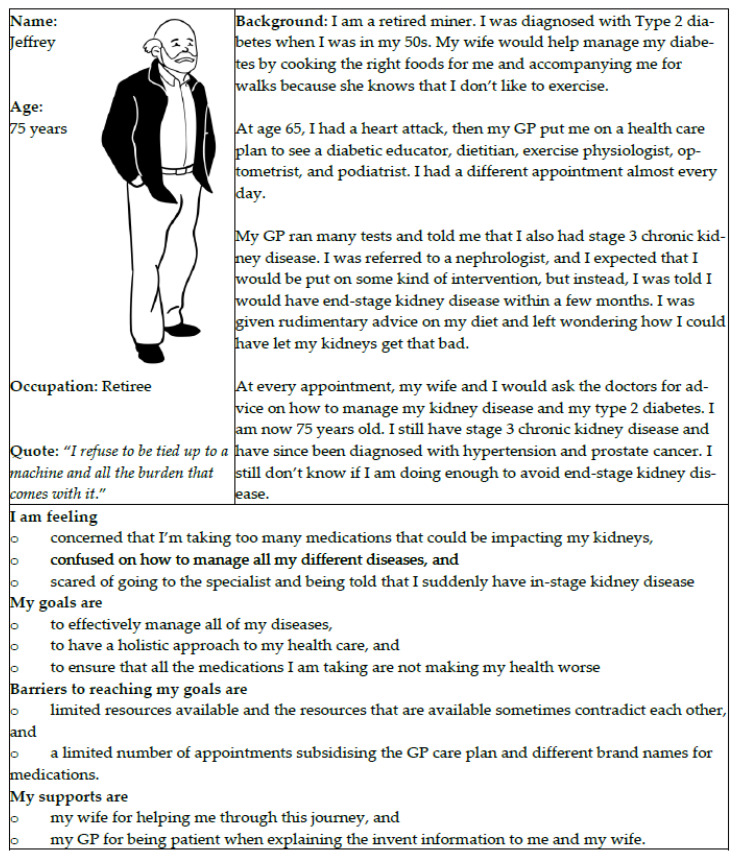
Example of a persona of an older CKD patient using a fictitious name. The persona is based on the patient’s personal experiences and background, emotions, desires, perceived barriers, and supports.

All users attending the workshops created their own personas using a fictitious name but based on their personal experiences and background, emotions, desires, barriers, and supports. In addition, users shared their own personas with other users within their small groups at the workshops. Users were also encouraged to specifically share their personal interactions with local health services. Their key insights were recorded on Post-it notes again by the external facilitator or researcher. The users then placed these Post-it notes back on the journey map wall chart, and a photograph was taken to enable all journey maps to undergo further analysis by researchers following the workshops. Researchers were also tasked with grouping similar individual personas to produce “aggregated personas” to reflect the different users engaging with Illawarra-Shoalhaven CKD services. Personas of individuals with CKD, their families and caregivers, healthcare providers (including medical doctors, general practitioners, nephrologists, nurses, and allied health professionals), and others were developed, each representing different perspectives. An example of a persona is shown in Figure 7.

### 3.3. Ideation Workshops

A few months after the *Empathy Workshops*, all users were invited back to a new round of workshops to ideate and brainstorm solutions or changes required to improve services and experiences for people living with CKD in the Illawarra-Shoalhaven region. Following similar methodologies as described for the *Empathy* workshops, a number of improvements to services were identified, enabling researchers to more accurately describe a potential future state for all users.

After the *Ideation* workshop, a pamphlet was produced to summarise the outcomes discussed (Supplementary Materials, File S1). To validate the contents of the pamphlet as an accurate reflection of users’ thoughts and feelings, face-to-face meetings were held with a sub-group of users who had attended the *Ideation* workshops, in their home or at the University of Wollongong. Any feedback or gaps were taken into account, and the pamphlet was updated. This cross-checking activity with end users reflected the project’s ongoing commitment to authentic co-design that aims to continually place the end user at the centre throughout the research process.

### 3.4. Interviews

Attendance at the workshops by primary and tertiary health care professionals was low due to work commitments. To overcome this gap, semi-structured interviews were conducted with a purposive sample of nephrologists, general practitioners, renal and general practice nurses, dietitians, and social workers, at a time that was convenient to them [36]. Design Thinking methodology encourages the employment of different tools in order to capture all stakeholders’ experiences and insights, and to be guided by their individual needs, so that pragmatic barriers such as non-attendance due to work commitments do not limit the representation of key stakeholders. Insights from the workshops proved valuable when constructing the question guides for use in the interviews, representing another advantage of the design-led approach that promotes cycles of iteration and refinement. The verbatim interview transcripts were analysed using a relativist ontological position and a directed content analysis approach by three independent researchers, who reached consensus on key themes. These themes confirmed that the delivery of person-centred care for people with CKD was important among all health care professionals; however, a deficit in shared understanding of the disease within and between disciplines was identified, and a major barrier to an integrated approach to delivery of health care to individuals living with early-stage kidney disease [36].

## 4. Discussion

In summary, data from discussions at empathy and ideation workshops and interviews afforded HIRC CKD researchers, alongside end-users, the opportunity to more meaningfully describe what a desirable future state for people living with CKD in the Illawarra-Shoalhaven region may look like. This future state would be inclusive of empathetic and personalised experiences through an integrated kidney care service, notably involving more seamless communication and patient management by primary, secondary, and tertiary health care providers. Specifically, the pamphlet summarising the ideation sessions, alongside findings emerging from all the different activities in workshops and interviews, will be used as collateral in future discussions with health service decision makers to encourage the redesign and re-imagination of kidney care services in the Illawarra-Shoalhaven region. This final step ensures that attention, respect, and empathy towards end users’ perspectives and recommendations for service improvements are fully afforded. 

Positioning the design-led chronic kidney disease case-study within a traditional theoretical research framework

**Figure 8:**
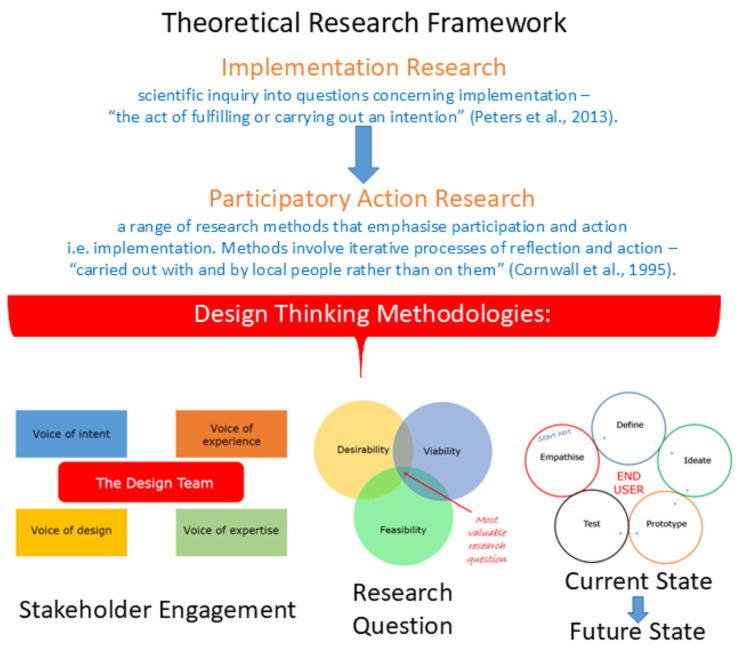
Conceptual schematic of the CKD Co-Design Project. This figure illustrates how the project was underpinned by Design Thinking methodologies and situated within the broader context of Implementation and Participatory Action Research, described by Peters et al. [45] and Cornwall et al. [46]. The schematic represents the integration of practical actions (e.g., problem definition, ideation, prototyping, iteration) with theoretical frameworks, highlighting how these approaches merge to guide the co-design process.

From an academic perspective, the HIRC CKD project falls under the high-level framework of “Implementation Research” (IR), a field of health research that has been shown to contribute to more effective public health, clinical policies, and programme designs [45] (Figure 8). IR is a scientific enquiry into questions concerning implementation or “the act of fulfilling or carrying out an intention” and is especially concerned with the users of the research, not merely the production of knowledge [45]. There is a wide range of implementation-specific research methods, including qualitative methods (grounded theory, ethnography, phenomenology, case studies and narrative approaches, key informant interviews, and focus groups), quantitative methods (cross-sectional surveys), and mixed methods (a combination of qualitative and quantitative methods) [45]. Specifically, the HIRC CKD project is underpinned by key principles of Participatory Action Research (PAR), a specific type of IR. PAR refers to a number of different research methods that emphasise participation and action (such as implementation), using methods that involve iterative processes of reflection and action, “carried out with and by the local people, rather than on them” [46]. In PAR, the control of the process rests with the participants, which includes people with CKD, their families/carers, health care practitioners, domain experts, and academics/researchers in the case of the proposed research project.

Improvements to traditional qualitative research, which purports to be “co-designed”, can be made through a commitment to applying the core principles of authentic Design Thinking. This means aiming to, firstly, conduct an intent meeting with end users and people who represent all four Design Voices at the start of a project to help clarify what the problem is and to define what the current state is, whilst pondering what the future state might look like. This helps to identify the gaps and gives some ideas about what changes might be necessary. It also helps to better define the problem statement and identify additional key stakeholders across the four Design Voices to ensure the best approach. It also means co-designing the research question rather than having researchers propose the question from the outset based on perceived gaps from previous literature or work. Finally, it means to always maintain representation of all four Design Voices throughout the duration of the project to minimise bias. Challenges with these recommendations may be the greater initial time investment, for example, even before the research question is proposed. From an ethics perspective, there may be concerns that stakeholders are approached to help define the project intent and research question prior to the research being approved by an ethics committee. Delineation between engagement prior to “official” research commencing may be required. Finally, because cycles of iteration are a core part of the design thinking process, research protocol changes may be more frequent, and this could have implications for ethics committees that require reporting and approval of any amendments. The precise nature of the expected “looping” and potential iterations should therefore be stated within original ethics applications to mitigate this. Increasingly, advocacy for authentic co-design is growing to empower both researchers and participants, service providers and service users, policy makers and community members [47].

### Limitations

While the design-led approach offers valuable opportunities to co-create meaningful health solutions, several limitations should be acknowledged. First, authentic co-design requires extensive stakeholder engagement across the four Design Voices. This can be resource- and time-intensive, often demanding substantial coordination before a formal research protocol is approved. Such requirements may not be feasible in all health research contexts, particularly where funding, staffing, or timelines are constrained. Second, the iterative and cyclical nature of Design Thinking can pose challenges for ethics approval processes, since frequent refinements to protocols and outputs may necessitate multiple amendments. This tension between the flexibility of Design Thinking and the rigidity of institutional governance frameworks can limit methodological agility. Third, participation bias may arise. Stakeholders who are more motivated, available, or vocal may be overrepresented, while harder-to-reach groups (e.g., people with advanced disease, carers with limited availability, or marginalised communities) may have less influence in shaping outcomes. This could inadvertently undermine the equity aims of co-design. Finally, translating co-designed ideas into sustainable health service change remains a challenge. Even when solutions are desirable and feasible, systemic constraints, such as workforce shortages, siloed care structures, and limited resources, may hinder their implementation at scale. Future research should therefore explore mechanisms to embed co-designed outputs into policy and service redesign in a sustainable manner.

## 5. Conclusions

This paper describes how a commitment to key principles of Design Thinking can more authentically achieve co-designed research outcomes, aiming to explore a complex health problem in the community. Using the case study of high CKD rates in the Illawarra and Shoalhaven regions of Australia, we showcased a design-led methodological approach that ensures end-users remain at the centre of all research throughout the project’s lifespan, from the conception of the research question and protocol to solution generation and change implementation. Representation of the four Design Voices with appropriate stakeholders throughout the research project is imperative to minimise bias towards researchers as the main research orchestrators. This paper showed that empathising through direct interaction with users is crucial to making sense and creating meaning of users’ problems and their challenges. Ideation ensures user-centred solution generation, which is likely to be closer to addressing the real problem and creating an improved future state. The future planned prototyping/testing phases for this project will offer further validation of the user-centred ideas and solutions. Through this case study, we encourage the incorporation of Design Thinking principles for solution generation in health research as a valuable adjunct to traditional qualitative research methodologies, with the potential to facilitate meaningful outcomes for people in our community experiencing a wicked health problem anywhere in the world.

## Data Availability

Data available on reasonable request.

## Supplementary Materials

The following supporting information can be downloaded at: https://www.mdpi.com/article/10.3390/ijerph22101475/s1. File S1: Workshop findings Report for participants.
