# Supplementary material for: Applying Design Thinking for Co-Designed Health Solutions: A Case Study on Chronic Kidney Disease in Regional Australia
Source: Int J Environ Res Public Health. 2025 Sep 24;22(10):1475. doi: 10.3390/ijerph22101475 (PMC12564462; doi:10.3390/ijerph22101475)
Supplement: Supplementary file 1 [file ijerph-22-01475-s001.zip › ijerph-3643600-supplementary.pdf]

Who we are

This project was led by researchers from the XXXX RESEARCH CLUSTER at the XXX and was approved by the XXX Human Ethics Committee.

Contact Us

XXXXXX

XXXXXX

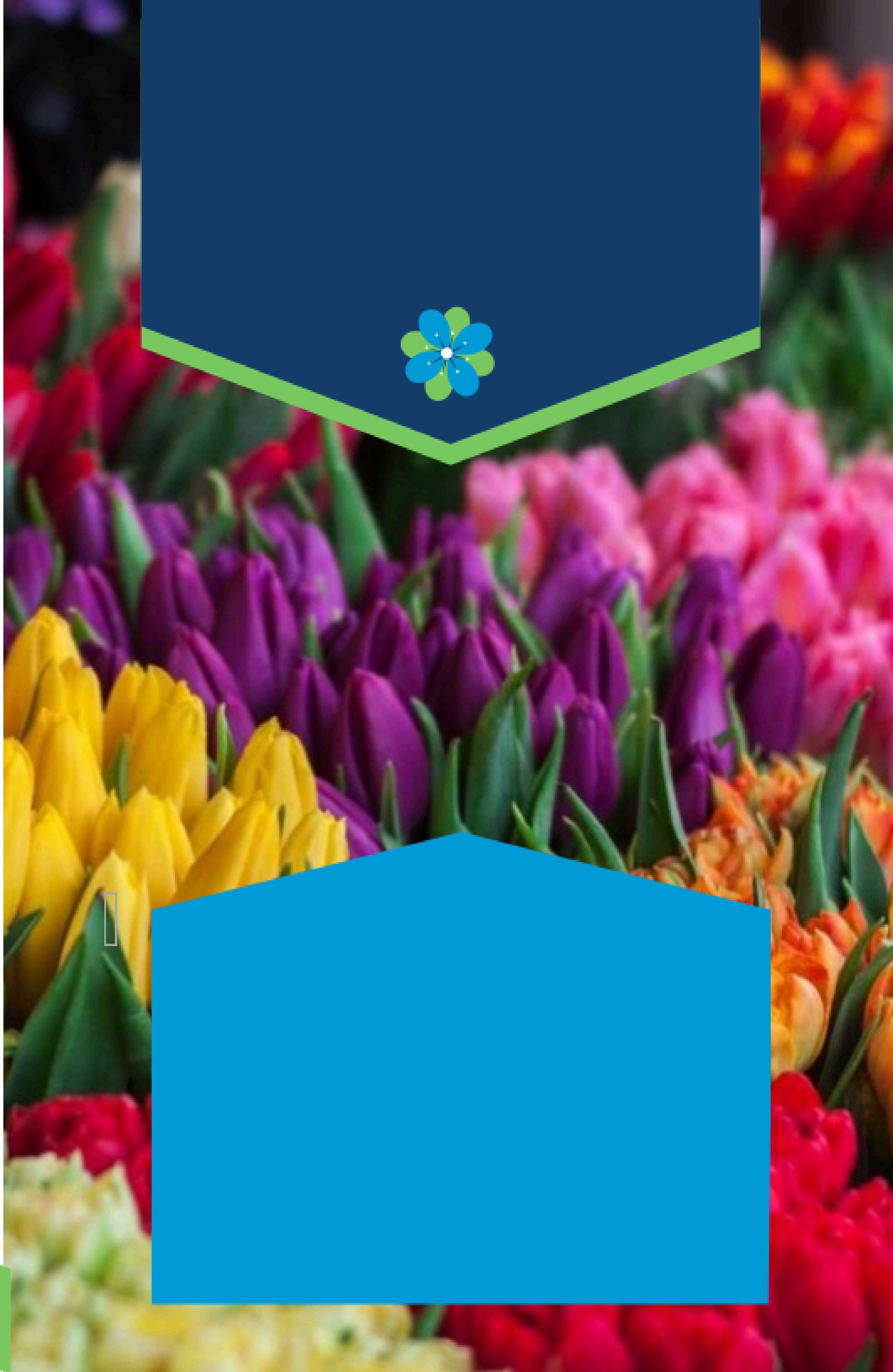

Living with

Chronic Kidney Disease

Thank you

The University of XXX, together with the XXX Local Health District, ran three workshops from September to November 2018. The workshops aimed to better understand the experiences of people living with chronic kidney disease, their families, carers and health care providers in the Illawarra Shoalhaven region.

Your personal stories at the workshops were very important in this project and helped to make it a great success.

We would now like to share with you some insights from the workshops in this draft booklet. We welcome any feedback you may have before we write the final copy.

Again, we warmly thank you for being a part of this great project!

Warm Regards,

The XXX Research Team

We now understand that:

Chronic kidney disease is like a puzzle

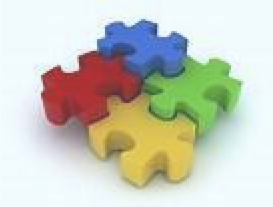

People experience chronic kidney disease (CKD) in different ways.

For some people, the disease takes a quick hold and then stays stable for a long time.

For others, not much changes even after 20 years.

In some cases, people start dialysis not long after they are diagnosed.

Chronic kidney disease is not straight forward, which may explain the different journeys that people experience.

This booklet sums up some of these unique experiences to help us better understand what it is like to live with chronic kidney disease in our local area.

People with chronic k[illegible]sease, and their carers, want advice about how the disease fits into their life

- People living with CKD described being told they have the disease without much hope:

“there will be 6 months until dialysis - do what you need to do in that time”

- Rather than a “dooms day prophecy”, people living with CKD want to be given all treatment options at the start **in a positive way**
- People feel **fear, shock** and **denial** when first receiving the diagnosis. It was described as “*scary and overwhelming* ” followed by “*it can’t happen to me* ” and “*she’ll be right* ”
- People living with CKD feel that is it important to have a carer present for support and to help interpret information they are given
- Carers feel it is important that all the people affected by the disease get information, so they can better support the patient:

“hard not having the answers for loved ones ”

- People living with CKD and their carers want to play a main role in decisions about their health

.

- They want health professionals to have a two-way conversation with them and not be talked ‘down’ to
- They want doctors to take the time to explain things clearly so they can take charge of their own ‘health journey’
- They want to ask questions

“more time with health professionals in the early days of diagnosis”

People living with CKD, and their carers, find it hard to understand information and find trusted sources

- People living with CKD and their carers want to better understand the disease and how to slow it down
- They want advice about how to live a normal life with the disease and what the future might look like for them
- They want messages that are easy to understand, not complicated medical language
- For non-dialysis patients, the General Practitioner (GP) is the main information provider but usually doesn’t have time to explain things
- People living with CKD often receive mixed messages from different health professionals
- Information also comes [illegible] the internet, specialists and pharmacy h[illegible]hat sources to trust

Living with

Chronic Kidney Disease

- Some people living with CKD have to do a lot of research themselves to make sense of all the information
- People starting dialysis early in their journey receive lots of information and support, more than people in earlier stages of CKD
- The Renal Unit is a great source of information, offering training, expert advice and a sense of community:

The Renal Unit provided "support for us to help take in the information" and "take the sting out of the situation"

- The **RENAL DIET** is difficult for people living with CKD and their carers to understand and follow
- They believe the diet is complicated, especially when they are also following another diet to manage a different condition, such as diabetes
- Specialist waiting rooms and the hospital environment can be very stressful and often trigger fears surrounding the disease. It was suggested that TV screens in the waiting rooms could be used to provide educational podcasts or information about research being done in the community, rather than entertainment TV

Invisible aspects of the disease present challenges for individuals and carers

- CKD is sometimes called an *INVISIBLE* disease because people often do not look sick. This can make it hard for them at work, at school or when using health services
- People living with CKD but not on dialysis, have a real fear of dialysis because they are afraid of losing their ability to work, to be active, to be good parents and to care for themselves. There is a fear of losing the ability to be self-determining and failing their family, with one individual explaining:

"I get tired and can't keep up with the kids"

- People having in-home dialysis describe it as '*injecting life*' and are happy with the support provided and have 'around the clock' telephone help
- Young adults living with CKD often feel they are not taken seriously:

"we have to convince people that there is a problem"

- People with CKD are not sure what support services are available to them (psychological/ educational/ social), particularly those who do not visit the Renal Unit

- Tapping into disability services for *home help* is very difficult. People need to persevere, which can be hard when you have a chronic condition. Younger people are often denied help
- Some people with CKD experience **depression** and feel as though no one understands what they are going through. It is easy for fear to take over:

"during the night, I am the sum of my failures", "a ticking time bomb"

- People with CKD and their carers describe many things they battle, and on some days, they just want to give up. Some have given up work and feel stressed and worried about how to support themselves and their families
- Living with CKD can be scary and lonely. Talking to other people with the disease helps to alleviate some fears. GPs seem to have more time to talk about mental health
- Support groups, either face-to-face or via social media, are considered valuable to help maintain good mental health to live a full and happy life
- People living with CKD and their carers believe that peer support could help to improve their self-worth:

"searching for someone who has been there, to see that there is hope"

- People living with CKD and their carers described how medications are often complicated and that even depression can be a side effect, as well as tiredness and an unsettled mind, which add to the difficulty in staying focussed and being able to break through the barriers to access education and support.
- Dialysis patients believe the Renal Unit is excellent and gives a lot of support, although the health professionals have less time to do what they need

Some patients say that dialysis is very restrictive and has "changed my life completely; I can't do what I want"

- Long distances from specialist services is a problem for people living in regional areas, making the travel tiring and frustrating

There is a need for the health system to care for the whole person and for health professionals to coordinate and collaborate more effectively across specialties

- Some people need to hear information a few times before it is understood and they want to feel supported to get tailored help
- Young people living with CKD have different needs and attitudes to the disease and feel misunderstood

Frustration that [illegible] in different specialties do not talk to each other to provide a "tailored diet for us as a whole person"

- People living with CKD and their carers feel there isn't good communication between the GP and specialists, and between specialists
- People living with more than one illness are confused about how treatments affect the different conditions – especially medications and diet

---

Some people feel that doctors who do not show compassion make them "feel like a punching bag"

- Having a number of conditions makes the CKD journey more complex. Waiting times for procedures can be lengthy and often cause the person living with CKD to be in physical pain and emotional stress for a long period of time

---

Having health professionals that listen is of high importance "you are the most important person to me right now.""

- Usually, specialist staff showed a high level of dedication to looking after patients

- Repetition of a health history to different health professionals is tiresome, with one person saying:

"I am starting to think I should carry a resume of my medical history"

- Some people living with CKD have had double blood tests and ultrasounds because specialists do not communicate with each other

Relationships with health professionals are all different and play a vital role in patients' attitudes to treatment

- Patients talk about the Renal Unit as a family, a place where they "*feel very loved and cared for*". People feel supported and heard by the Renal Unit staff
- Young carers and people with kidney disease express frustration that they are often not taken seriously, that their story has to be retold. They feel a lot of time is needed to build trust and to be heard

---

Carers believe that people living with CKD sometimes act differently in front of the doctor, to "keep the doctor happy"

People living with CKD and their carers "want to be heard and respected for [their] experience and knowledge"

- People with CKD describe that they can end up in hospital for the same reason (infection, for example), so they have a solid understanding of what is going wrong. However, they do not feel as though their opinion holds any weight in the minds of the health professionals, explaining that it's "*like I've never been in this situation before, when I have*"
- Regular doctor and dietitian visits help some people with CKD to stay on track with healthy lifestyle and diet choices

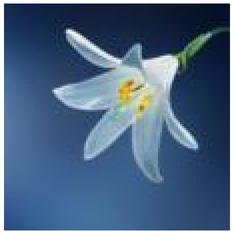

Living with

Chronic Kidney Disease
